# Synthesis, Docking Studies and Biological Evaluation of Benzo[*b*]thiophen-2-yl-3-(4-arylpiperazin-1-yl)-propan-1-one Derivatives on 5-HT_1A_ Serotonin Receptors

**DOI:** 10.3390/molecules17021388

**Published:** 2012-02-03

**Authors:** Hernán Pessoa-Mahana, Gonzalo Recabarren-Gajardo, Jenny Fiedler Temer, Gerald Zapata-Torres, C. David Pessoa-Mahana, Claudio Saitz Barría, Ramiro Araya-Maturana

**Affiliations:** 1 Departamento de Química Orgánica y Fisicoquímica, Facultad de Ciencias Químicas y Farmacéuticas, Universidad de Chile, Casilla 233, Santiago 1, Chile; 2 Departamento de Farmacia, Facultad de Química, Pontificia Universidad Católica de Chile, Casilla.306, Santiago 22, Chile; 3 Departamento de Bioquímica y Biología Molecular, Facultad de Ciencias Químicas y Farmacéuticas, Universidad de Chile, Casilla 233, Santiago 1, Chile; 4 Unidad de Gráfica Molecular, Departamento de Química Inorgánica y Analítica, Facultad de Ciencias Químicas y Farmacéuticas, Universidad de Chile, Casilla 233, Santiago 1, Chile

**Keywords:** arylpiperazines, benzo[*b*]thiophene, depression, 5-HT_1A_ receptor, docking, microwave Michael addition

## Abstract

A series of novel benzo[*b*]thiophen-2-yl-3-(4-arylpiperazin-1-yl)-propan-1-one derivatives **6a–f**, **7a–f** and their corresponding alcohols **8a–f** were synthesized and evaluated for their affinity towards 5-HT_1A_ receptors. The influence of arylpiperazine moiety and benzo[*b*]thiophene ring substitutions on binding affinity was studied. The most promising analogue, 1-(benzo[*b*]thiophen-2-yl)-3-(4-(pyridin-2-yl)piperazin-1-yl)propan-1-one (**7e**) displayed micromolar affinity (*K_i_* = 2.30 μM) toward 5-HT_1A_ sites. Docking studies shed light on the relevant electrostatic interactions which could explain the observed affinity for this compound.

## 1. Introduction

Serotonin (5-hydroxytryptamine, 5-HT), is an important neurotransmitter that plays a role in regulating numerous physiological functions, including thermoregulation, vasoconstriction, sexual behavior, appetite and sleep [1,2,3]. Additionally, it has been consistently implicated in the pathophysiology of a number of psychiatric disorders such as depression and anxiety. The 5-HT selective reuptake inhibitors (SSRIs), are currently the first line therapy for depression. However, a serious drawback in the treatment by SSRIs is on one hand, the delay of therapeutic benefits believed to be caused by the 5-HT_1A_ auto receptors’ inhibitory role, and on the other, the lack of selectivity of the obtained compounds. For these reasons efforts in developing newer antidepressants for a fast acting agent are still ongoing [4,5]. Among the various 5-HT receptors, the 5-HT_1A_R has been the most extensively studied, as a result, ligands acting as partial agonists on this subtype have demonstrated effectiveness in the treatment of anxiety and depression [6,7].

During the last twenty years several chemical scaffolds are known to bind 5-HT_1A_R sites, among these, arylpiperazine derivatives represent one of the most studied skeletons [8,9,10].

**Figure 1:**
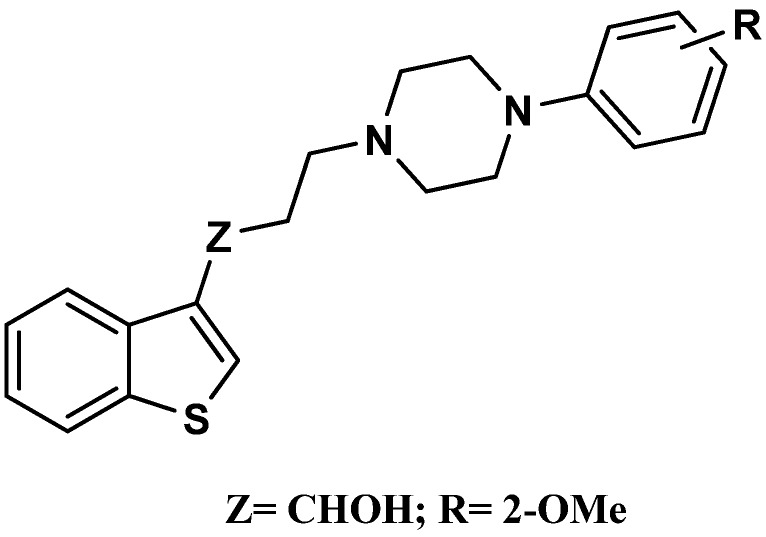
General structure of benzothiophene hydroxylpropylarylpiperazine series.

In the search for new antidepressants, Monge *et al*. reported the synthesis and biological evaluation of a series of unsubstituted benzothiophene derivatives connected at C-3 with subtituted aryl-piperazines by a polymethylenic chain (Figure 1). The best pharmacological activities were obtained with the benzothiophene hydroxylpropylarylpiperazine series, with the highest 5-HT_1A_R affinity (*K*_i_ = 20 nM) being observed for the 2-methoxyphenylpiperazine derivative [11,12].

**Figure 2:**
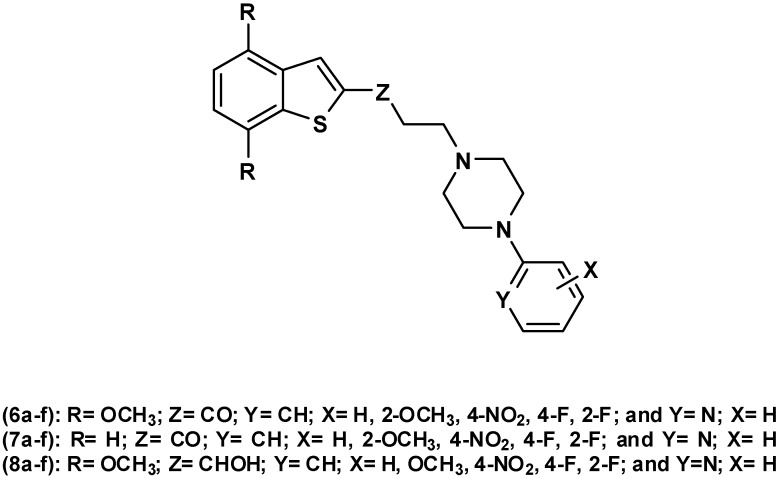
Series of benzo[*b*]thiophene compounds synthesized in this study.

Recently, Perez-Silanes *et al.*[13] reported the synthesis and binding assays on SERT and 5-HT_7_R of a series of benzo[*b*]tiophen-2-yl-propenones connected to arylsulfonamide moieties, which displayed a rapid onset of action. Given our interest in this field, a new set of benzo[*b*]thiophenyl arylpiperazinyl propan-1-one derivatives was synthesized, in order to study the influence of a novel C-2 substitution pattern on 5-HT_1A_R binding affinity. It is expected that this new C-2 substitution pattern affects not only the electronic density on the benzo[*b*]thiophene moiety, but might provide compounds with improved 5-HT_1A_R affinities. The synthesized series are summarized in Figure 2.

In a previous work, we reported a versatile approach for obtaining novel benzothiophene bis-ligands using solvent-free microwave assisted aza-Michael addition, connecting functionalized arylpiperazines to benzo[*b*]tiophen-2-yl-propenones. This strategy is used as the base reaction in the present study [14,15].

With the aim of elucidating probable binding modes of these compounds upon binding to 5-HT_1A_R, molecular modeling and docking studies of substituted 2-[oxoalkyl](4-arylpiperazinyl)]benzo[*b*]-thiophene were carried out using an homology model obtained from the β_2_-adrenergic crystal structure deposited in the Protein Data Bank [16].

## 2. Results

### 2.1. Chemistry

**Scheme 1:**
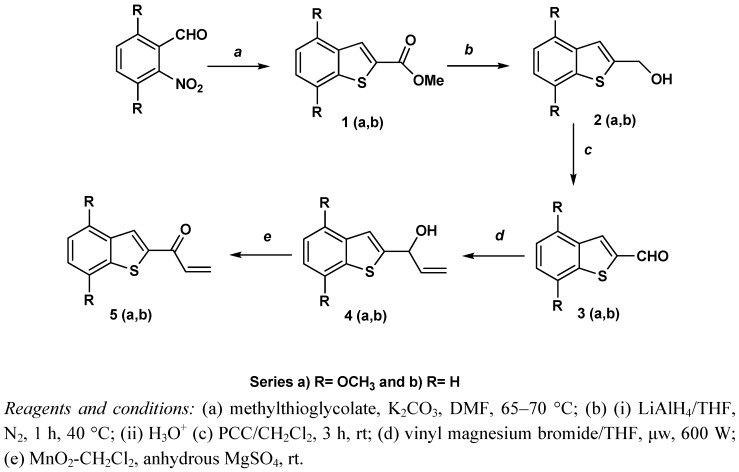
Synthetic approach used to obtain 1-(benzo[b]thiophen-2-yl)prop-2-en-1-ones **5a–b**.

In an effort to obtain compounds with improved affinities, eighteen new benzothiophene arylpiperazines were synthesized and their bioactivities evaluated on 5-HT_1A_R. The presence of ketopropyl and hydroxypropyl spacers as connecting chains between the benzothiophene and arylpiperazine moieties, along with the introduction of substituted aromatic rings at the N-4 piperazine atom, were performed. The synthetic approach for the preparation of the target compounds, benzothiophene 2-propenones **5a**,**b**, (series a: R = OCH_3_; series b: R = H) is outlined in Scheme 1. The general procedure is as follows: benzothiophene aldehydes **3a**,**b** were prepared in three steps, starting from 2,5-dimethoxy-6-nitrobenzaldehyde and commercially available 2-nitrobenzaldehyde in presence of methylthioglycolate to provide esthers **1a** and **1b** in 72% and 75% yields, respectively. The esters were reduced with LiAlH_4_ to afford the alcohols **2a**,**b**, with yields of 65% and 71%, respectively.

The synthesis of the key ketones **5a**,**b** was carried out by a microwave supported Grignard reaction of the corresponding benzo[*b*]thiophene-2-carboxaldehydes **3a**,**b** with vinyl magnesium bromide, followed by oxidation of the corresponding alcohols **4a**,**b** with MnO_2_ to give ketones **5a**,**b** in relatively good yields of 58% and 65%, respectively.

**Scheme 2:**
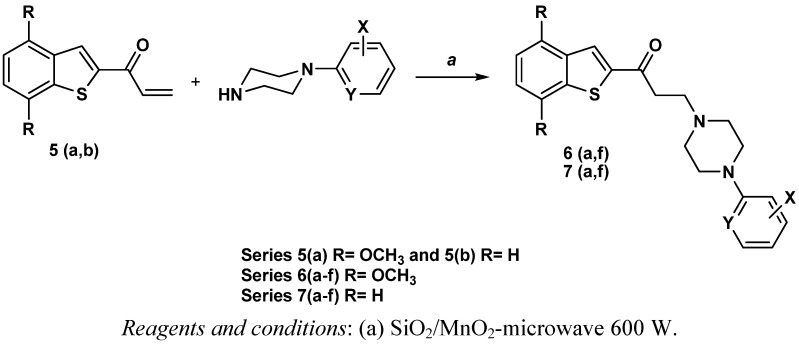
Synthesis of series **6a–f** and **7a–f** under microwave conditions.

The ketoarylpiperazine derivatives **6a–f** and **7a–f** were prepared by Michael addition of ketones **5a**,**b** with different arylpiperazines using free solvent microwave irradiation conditions according to Scheme 2. Melting points and reaction yields for these compounds are reported in Table 1.

**Table 1:** Series (**6a–f** and **7a–f**) obtained through Michael addition reaction between arylpiperazines and benzo[*b*]thiophenpropenone **5a**,**b**.

Entry	Y	X	R	Yield (%)	m.p (°C)	Entry	Y	X	R	Yield (%)	m.p (°C)
**6a**	CH	H	OMe	88	169–170	**7a**	CH	H	H	59	….. ^a^
**6b**	CH	2-F	OMe	66	134–135	**7b**	CH	2-F	H	90	….. ^b^
**6c**	CH	4-F	OMe	60	131–132	**7c**	CH	4-F	H	38	117–118
**6d**	CH	2-OMe	OMe	67	128–129	**7d**	CH	2-OMe	H	50	45–47
**6e**	N	H	OMe	60	174–175	**7e**	N	H	H	71	56–58
**6f**	CH	4-NO_2_	OMe	65	161–162	**7f**	CH	4-NO_2_	H	69	138–141

^a,b^ yellow oils.

**Scheme 3:**
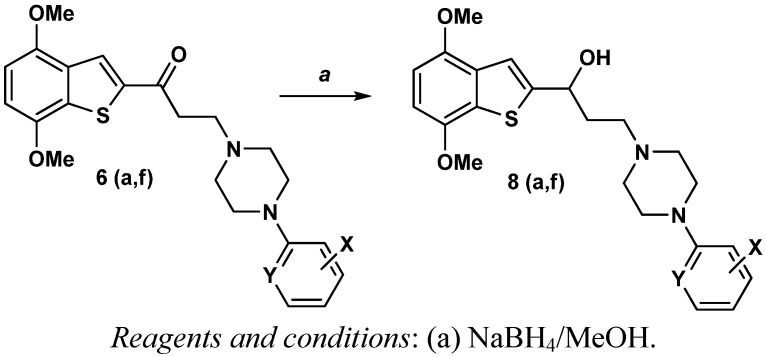
Synthesis of the reduced derivatives **8a–f**.

The corresponding hydroxyl derivatives **8a–f** depicted in Scheme 3 were obtained in good yields (Table 2) upon treatment of the ketoarylpiperazine derivatives **7a–f** with sodium borohydride in methanol.

**Table 2:** Yields of the reduced derivatives **8a–f**.

Entry	Y	X	R	Yield (%)	m.p (°C)
**8a**	CH	H	OMe	68	148–149
**8b**	CH	2-F	OMe	63	128–129
**8c**	CH	4-F	OMe	80	55–56
**8d**	CH	2-OMe	OMe	93	55–56
**8e**	N	H	OMe	77	157–158
**8f**	CH	4-NO_2_	OMe	72	173–174

### 2.2. Biological Properties

Target compounds **6a–f**, **7a–f**, and **8a–f** were assessed for *in vitro* affinity on serotoninergic 5-HT_1A_R by radioligand binding assays, using [^3^H]-8-OH-DPAT in rat cerebral cortex membranes. All the compounds were DMSO soluble as bases. Due to practical considerations, in the experimental binding assays, these compounds were first tested at a fixed dose of 1 μM, and only for those that in the screening process showed a displacement of the radioligand of ≥55%, the dose-response curves were determined. The inhibition constant *K*_i_ was calculated from its IC_50_ value only for compound **7e**, using the Cheng-Prusoff equation The results of these assays are summarized in Table 3. The 8-OH-DPAT displayed an inhibition percentage of 92.

**Table 3:** Inhibition % for benzo[*b*]thiophene arylpiperazine derivatives.

Entry	Y	X	R	Inhibition (%)	Entry	Y	X	R	Inhibition (%)
**6a**	CH	H	OMe	2 ± 2	**7-d**	CH	2-OMe	H	52 ± 7
**6b**	CH	2-F	OMe	14 ± 2	**7-e**	N	H	H	60 ± 4
**6c**	CH	4-F	OMe	0 ± 3	**7-f**	CH	4-NO_2_	H	0 ± 4
**6d**	CH	2-OMe	OMe	44 ± 2	**8-a**	CH	H	OMe	24 ± 4
**6e**	N	H	OMe	17 ± 5	**8-b**	CH	2-F	OMe	21 ± 1
**6f**	CH	4-NO_2_	OMe	0 ± 3	**8-c**	CH	4-F	OMe	23 ± 3
**7a**	CH	H	H	…… ^a^	**8-d**	CH	2-OMe	OMe	38 ± 0
**7b**	CH	2-F	H	33 ± 3	**8-e**	N	H	OMe	27 ± 3
**7c**	CH	4-F	H	31 ± 7	**8-f**	CH	4-NO_2_	OMe	14 ± 2

^a^ not measured.

All the synthesized compounds displayed inhibition with regards to [^3^H]-8-OH-DPAT binding, with percentages ranging from 0% (compounds **6c**, **6f** and **7f**) to 60%, corresponding to compound 1-(benzothiophen-2-yl)-3-(4-(pyridin-2-yl)piperazin-1-yl)propan-1-one (**7e**). Consequently, a dose-response curve was plotted out using **7e**, under the same pH conditions, temperature and time as stated in the Experimental section. Values for **7e** concentrations encompassed six orders of magnitude (1 nM–1 mM), thus, its obtained IC_50_ and K_i_ values were 2.50 and 2.30 μM, respectively.

Focusing our aims toward to the exploration of new molecules endowed with 5-HT_1A_ activity, binding values for compounds **6a–f**, **7a–f** and **8a–f** were analyzed in order to examine the influence of functionalized arylpiperazines linked to benzo[*b*]thiophene moieties at C-2.

### 2.3. Docking Studies

#### 2.3.1. Molecular Modeling Studies of 5-HT_1A_R in complex with Ligands **6d**, **6f**, **7f**, **7e**

In order to get a better understanding of the benzo[*b*]thiophene arylpiperazine receptor interactions, computational simulations were carried out using an homology model for the 3D structure of 5-HT_1A_R.

From the pharmacological results, the pairs **6d/6f** and **7f/7e** displayed the lowest and the highest binding affinities in these series, respectively. These results were interpreted according the interactions obtained in the docking studies. The results are summarized in Table 4.

**Table 4:** Displayed interactions in the binding site.

Residues 5-HT_1A_R	Ligands			
	6d	6f	7f	7e
D116	+	+	+	+, ¥
F360	Y	Y	Y	Y_a_
F361	Y	-	-	NOI
L380	NOI	-	-	+
N385	NOI			

+: H-bonding; Y: Hydrophobic interactions; Y_a_: Edge-to-face interactions; -: Repulsive interactions; ¥: electrostatic interactions; NOI, no observed interactions.

The docking results were analyzed according to the structures with the best total energies, displaying the shortest H-bond between the aspartic residue D116 and the basic protonated nitrogen of the piperazine ring of the ligands. The electrostatic and hydrophobic interactions observed in the complexes were also considered.

**Figure 3:**
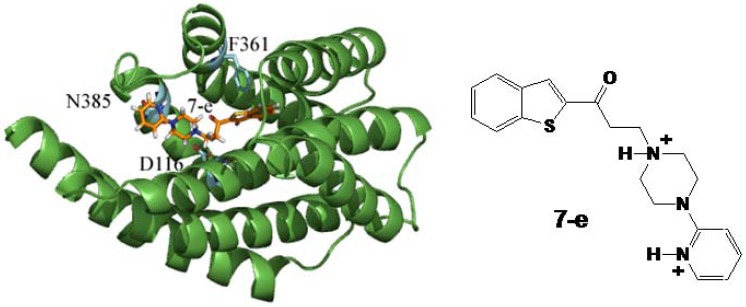
Compound **7e** docked to 5-HT_1A_ molecular model receptor. Interacting residues with compound **7e** are shown in bold.

As mentioned in Section 2.2, compound **7e** displayed the best binding profile in the radioligand studies with 60% of inhibition (Table 3) (*K_i_* 2.30 μM). This result was interpreted according to the docking criteria described above and considering a diprotonated form of compound **7e** as represented in Figure 3, which shows the molecular docking of compound **7e** in its preferred conformation at the binding site of 5-HT_1A_R. The observed interactions were an H-bond between D116 and protonated N-1 at a distance of 2.83 Å, reinforced approximately by 10 kcal/mol product by the electrostatic interaction resulting between the aspartate moiety and the N-1 quaternary ammonia of the piperazine ring [17].

Another H-bond was observed at 3.19 Å between the protonated nitrogen of the pyridine ring and the carbonyl amide group of the side chain of N385 reinforced by an ion-dipole at 2.95 Å. The stabilization of the latter interactions may vary energetically among 1–7 kcal/mol [16]. These interactions might explain the observed affinity of compound **7e** obtained in the binding assays. This complex also displayed edge-to-face aromatic-interactions [18] between the F361 and the benzo[*b*]thiophene moiety of the ligand. These weak attractive interactions arise among aromatic groups that do not necessarily bear polar substituents; however, they play an important role in protein folding, ligand-binding, supramolecular complexes and drug-receptor interactions [18].

On the other hand, the model complex for **7f** (displaying no activity), shows an H bond, at a distance of 3.01 Å between the N-1 and D116 along with repulsive interactions between the *p*-nitro group of the aromatic ring linked to piperazine with L380 at a distance of 2.79 Å, and the residue N385 at a distance of 2.89 Å. This suggests that groups bearing strong electron-withdrawing features decrease the affinity of the ligand for the binding site which can be attributed to two factors: one of electronic nature and other due to an unfavorable steric hindrance of the nitro group in that position. Hindrance effects have been also observed in the binding properties to the 5-HT_1A_ receptors of structural related ligands such as coumarins [19]. Thus, it would be reasonable to think that these repulsive interactions are essential in the observed null activity for both compounds **6f/7f**, confirming that voluminous groups at *p*-position of the aromatic ring linked to piperazine are detrimental for the proper affinity with the receptor.

Comparing these four ligand-receptor complexes, we conclude that several electrostatic or hydrophobic in nature stabilizing interactions are present. The molecular docking of series **6d**, **6f**, **7e** and **7f** showed that they bind to the 5-HT_1A_R in a similar fashion, being the common feature for these compounds the presence of a H-bond between the D116, and the protonated nitrogen of the piperazine ring with distances lower than 3.00 Å. However, the structural determinants for compounds showing measurable activity seem to be the absence of repulsive interactions, which can define in a greater extent the ability of the ligand to accomadate itself in the binding site.

Another compound that did not display any inhibitory activity was the *p*-fluoro compound **6c**. This fact reinforces the idea that an electronegative substituent in *p*-position of the phenyl ring does not favor the activity. However, the decreased volume of fluorine atom compared to the nitro group indicates that this lack of activity could be attributed to other structural features such as the presence of methoxy groups on the benzo[*b*]thiophene ring. According to this, *p*-F derivatives **8c** and **7c** displayed certain level of activity, with inhibition percentages of 23% and 31%.

The evaluated compounds belonging to the **8a–f** family showed better inhibition assays compared to their carbonylic analogues **6a–f**, suggesting that hydroxyl groups might have some influence in the affinity for the 5-HT_1A_R. This may be due to the hydroxyl participation in H-bonding interactions, stabilizing the ligand-receptor complexes or changes in volume and orientation (in terms of bond length and angles) underwent by the carbon atom bearing the hydroxyl group, when the hybridization goes from sp^2^ to sp^3^. These results are in agreement with the report by Martinez [20] for a series of benzo[*b*]thiophene derivatives, where the carbonyl compouds were compared with their respective alcohol derivatives.

On the other hand, the carbonylic derivatives belonging to family **7a–f** displayed an improved binding pattern compared to their structural analogues **6a–f**, probably due to the lack of voluminous methoxyl groups on the benzothiophene ring, which might have a detrimental effect on the affinity displayed. In this sense, the existence or absence of these groups in such compounds probably represent the most important structural determinants for their displayed activity. The decreased activity, going from derivatives **6e** (17%), **6b** (14%) and **6c** (0%) to their respective analogues without the methoxyl groups **7e** (60%), **7b** (33%) and **7c** (31%), confirm this statement. A fact that confirms this observation is that two compounds with the best competition profiles do not bear methoxyl groups in their scaffolds, namely compounds **7d** and **7e**. Within compounds that displayed better inhibition profiles, three of them, namely **6d** (44%); **8d** (38%) and **7d** (52%) bear a methoxy group at the *ortho *position of the aromatic ring linked to piperazine, strongly suggesting that the presence of this group represents a relevant and important characteristic for their increased activity. These results are in good agreement with previous studies, indicating that incorporation of *o*-methoxy groups lead to arylpiperazine compounds with higher affinities for 5-HT_1A_ receptors [21,22,23,24,25,26] and 5-HT_7_[26].

## 3. Experimental

### 3.1. General

Melting points were determined on a hot-stage apparatus and are uncorrected. The IR spectra were recorded on a FT-IR Bruker IFS 55 spectrophotometer for KBr discs and wavenumbers are reported in cm^−1^. The ^1^H-NMR and ^13^C-NMR spectra were recorded on a Bruker DRX-300 spectrometer (at 300 and 75 MHz, respectively) in deuterochloroform, or DMSO-d_6_. Chemical shifts were recorded in ppm (δ) relative to TMS as an internal standard. *J* values are given in Hz. The following multiplicity abbreviations were utilized: s = singlet, bs = broad singlet; d = doublet; dd = doublet of doublet; td = triplet of doublet; m = multiplet. The microwave assisted procedures were carried out in a Milestone, Lavis 1000 Multiquant. High resolution mass spectrum were recorded on a Thermo Finnigan model MAT 95XP Mass Spectrometer. Silica gel Merck 60 (70–230 mesh) and DC-alufolien 60 F_254_ were used for column and TLC chromatography, respectively. Purification of each product was carried out by chromatography on a silica gel column and/or recrystallized from the appropriate solvent.

### 3.2. Synthesis

*1-(4,7-Dimethoxybenzo[b]thiophen-2-yl)-2-propen-1-one *(**5a**) [14,15]. To a solution of alcohol **4a** (350 mg, 1.40 mmol) in CH_2_Cl_2_ (60 mL) was added MnO_2_ (365 mg, 4.20 mmol), and anhydrous MgSO_4_ (168 mg, 1.40 mmol), and the mixture stirred at room temperature for 3 h. The reaction mixture was filtered and the solvent removed under reduced pressure to give 250 mg, of crude enone **5a**. The crude residue was purified by column chromatography on silica gel (CH_2_Cl_2_) and recrystallized from (EtOH/cyclohexane 2:1) to afford pure **5a** (218 mg, 63%) as a yellow-orange solid. mp 99–100 °C; IR_νmax_ (KBr): 3054 (C-H Ar), 2968 (C-H Aliph.), 1665 (C=O), 1556 (C=C) cm^−1^; ^1^H-NMR (CDCl_3_): 3.94 (s, 3H, Ar-OMe), 3.96 (s, 3H, Ar-OMe), 5.92 (dd, 1H, 3′-H, *J *= 10.4 Hz and *J* = 1.6 Hz), 6.55 (dd,1H, 3′-H, *J *= 17 Hz and *J *= 1.6 Hz), 6.67 (d, 1H, 5-H, *J* = 8.5 Hz), 6.80 (d, 1H, 6-H, *J* = 8.5 Hz), 7.22 (dd, 1H, 2′-H, *J *= 17 Hz and *J* = 10.4 Hz), 8.16 (s, 1H, 3-H); ^13^C-NMR (75 MHz, CDCl_3_): δ 55.8, 56.0, 104.6, 107.6, 127.0, 129.6, 131.4, 131.6, 133.7, 143.6, 148.6, 150.8, 183.8; HRMS (EI) calcd for C_13_H_12_O_3_S (M^+^): 248.05072, found: 248.05001.

#### 3.2.1. General Procedure for the Synthesis of *1-(4,7-Dimethoxybenzo[b]thiophen-2-yl)-3-(4-arylpiperazin-1-yl)-1-propanone Derivatives* **6a–f**

*1-(4,7-Dimethoxybenzo[b]thiophen-2-yl)-3-(4-phenylpiperazin-1-yl)-1-propanone *(**6a**). To a solution of 1-(4,7-dimethoxybenzo[b]thiophen-2-yl)-2-propen-1-one (**5a**, 100 mg, 0.40 mmol) and 1-phenylpiperazine (93 mg, 0.57 mmol), in dichloromethane (20 mL) was added the supported inorganic reagent (MnO_2_–SiO_2_, 4:1, 1.0 g) and the suspension was vigorously stirred for 15 min at room temperature. The solvent was removed *in vacuo* and the solid was irradiated in a microwave oven at 900 W for 15 min. until TLC showed that the starting product had disappeared. The solid was thoroughly washed with AcOEt followed by remotion of the solvent to afford **7a** as a crude in quantitative yield. The solid residue was purified by crystallization (EtOH/CH_3_CN 5:1), to give the title compound as pale yellow crystals (138 mg, 88% yield); mp 169–170 °C; IR (KBr): 3032 (C-H Ar), 2934 (C-H Aliph.), 1655 (C=O), 1600 (C=C) cm^−1^; ^1^H-NMR (CDCl_3_): δ 2.71 (t, 4H, 2′′-H and 6′′-H *J *= 5.0 Hz), 2.95 (t, 2H, 3′-H, *J* = 7.4 Hz), 3.22 (t, 2H, 3′′-H, 5′′-H, *J *= 5.0 Hz), 3.24 (t, 2H, 2′-H, *J *= 7.4 Hz,), 3.95 (s, 3H, Ar-OMe C-4 or C-7), 3.96 (s, 3H, Ar-OMe C-7 or C-4), 6.68 (d, 1H, 5-H, *J *= 8.4 Hz), 6.8 (d, 1H, 6-H, *J *= 8.4 Hz), 6.87 (t, 1H, 4′′´-H, *J *= 7.4 Hz), 6.94 (d, 2H, 2′′′-H, 6′′′-H, *J *= 8.5 Hz), 7.27 (t, 2H, 3′′′-H and 5′′′-H, *J *= 8.3 Hz), 8.15 (s, 1H, 3-H); ^13^C-NMR (75 MHz, CDCl_3_): δ 36.7, 49.1, 53.1, 53.3, 55.8, 56.1, 104.6, 107.4, 116.1, 119.73, 129.1, 126.5, 131.5, 133.4, 142.8, 148.6, 150.7, 151.3, 193.3; HRMS (EI) Calcd for C_23_H_26_N_2_O_3_S (M^+^): 410.16641, found: 410.16618.

*3-[4-(2-Fluorophenyl)piperazin-1-yl]-1-(4,7-dimethoxybenzo[b]thiophen-2-yl)-1-propanone *(**6b**).Prepared from **5a** (50 mg, 0.20 mmol) and 1-(2-fluorophenyl)piperazine (55 mg, 0.31 mmol). Quantitative crude yield. Purified by recrystallization (EtOH/hexane 2:1), to give yellow crystals (53.6 mg, 65.7%); mp 134–135 °C; IR*_νmax_* (KBr): 3032 (C-H Ar), 2937 (C-H Aliph.), 1665 (C=O), 1500 (Ar C=C) cm^−1^; ^1^H-NMR (CDCl_3_): δ 2.72 (t, 4H, 2′′-H and 6′′-H, *J *= 4.8 Hz), 2.95 (t, 2H, 3′-H, *J* = 7.6 Hz), 3.12 (t, 4H, 3′′-H, 5′´-H, *J *= 4.8 Hz ), 3.23 (t, 2H, 2′-H, *J *= 7.6 Hz), 3.93 (s, 3H, Ar-OMe C-4 or C-7), 3.95 (s, 3H, Ar-OMe C-7 or C-4), 6.67 (d, 1H, 5-H, *J *= 8.5 Hz), 6.78 (d, 1H, 6-H, *J *= 8.5Hz), 6.90–7.09 (m, 4H, 3′′′-H, 4′′′-H, 5′′′-H, 6′′′-H), 8.14 (s, 1H, 3-H); ^13^C-NMR (CDCl_3_): δ 36.7, 50.5, 50.6, 53.2, 53.3, 55.8, 56.1, 104.6, 107.4, 116.1(d, *^2^J* = 21 Hz), 118.9.0 (d, *^3′^J* = 3.0 Hz), 122.5 (d, *^2′^J* = 8.0 Hz), 124.5 (d, *^4^J* = 3.6 Hz), 126.5, 131.5, 133.4, 140.1 (d, *^3^J* = 8.6 Hz), 143.0, 148.6, 150.8, 155.8 (d, *^1^J* = 245 Hz), 193.4; HRMS (EI) Calcd for (M^+^): C_23_H_25_FN_2_O_3_S 428.15699, found: 428.15686.

*3-[4-(4-Fluorophenyl)piperazin-1-yl]-1-(4,7-dimethoxybenzo[b]thiophen-2-yl)-1-propanone *(**6c**). Prepared from **5a** (70 mg, 0.282 mmol) and 1-(4-fluorophenyl) piperazine (77 mg, 0.427 mmol). Crude yield 96%. Purified by recrystallization (EtOH) as yellow pale crystals (73 mg, 60%); mp 131–132 °C; IR*_ νmax_* (KBr): 3032 (C-H Ar), 2940 (C-H Aliph.), 1655 (C=O), 1514 (Ar C=C) cm^−1^; ^1^H-NMR (CDCl_3_): δ 2.68 (t, 4H, 2′′-H and 6′′-H, *J *= 4.9 Hz), 2.92 (t, 2H, 3′-H, *J* = 7.5Hz), 3.11 (t, 4H, 3′′-H, 5′′-H, *J *= 4.9 Hz ), 3.21 (t, 2H, 2′-H, *J *= 7.5 Hz), 3.92 (s, 3H, Ar-OMe C-4 or C-7), 3.94 (s, 3H, Ar-OMe C-7 or C-4), 6.65 (d,1H, 5-H, *J *= 8.5 Hz), 6.77 (d, 1H, 6-H, *J *= 8.5 Hz), 6.82–6.98 (m, 4H, Ar-F (2′′′-H, 3′′′-H, 5′′′-H, 6′′′-H), 8.13 (s, 1H, 3-H); ^13^C-NMR (CDCl_3_): δ 36.8, 50.1, 53.2, 53.3, 55.8, 56.1, 104.6, 107.4, 115.5 (d, *^2^J* = 22 Hz), 117.8.(d, *^3^J* = 7.6 Hz),126.5, 131.5, 133.4, 140.1 (d, *^3^J* = 8.6 Hz), 143.0, 148.0 (d, *^4^J* = 2.2 Hz), 148.6, 150.8, 157.1 (d, *^1^J* = 239 Hz), 193.3 (C=O); HRMS (EI) Calcd for C_23_H_25_FN_2_O_3_S (M^+^): 428.15699, found: 428.15584.

*1-(4,7-Dimethoxybenzo[b]thiophen-2-yl)-3-[4-(2-methoxyphenyl)piperazin-1-yl]-1-propanone *(**6d**). Prepared from **5a** (53 mg, 0.21 mmol) and 1-(2-methoxyphenyl)piperazine (58 mg, 0.30 mmol). Crude yield 95.5%. Recrystallization from EtOH/hexane 5:1) gave pale white crystals (60 mg, 67% yield); mp 128–129 °C; IR*_νmax_* (KBr): 3031 (C-H Ar), 2940 (C-H Aliph.), 1663 (C=O), 1597 (C=C) cm^−1^; ^1^H-NMR (CDCl_3_): δ 2.75 (t, 4H, 2′′-H and 6′′-H, *J *= 4.4 Hz), 2.95 (t, 2H, 3′-H, *J* = 7.7 Hz), 3.31 (m, 2H, 3′′-H, 5′′-H), 3.27 (t, 2H, 2-H, *J *= 7.7 Hz), 3.87 (s, 3H, 2′′′-Ar-OMe), 3.94 (s, 3H, Ar-OMe C-4 or C-7), 3.95 (s, 3H, Ar-OMe C-7 or C-4), 6.67 (d, 1H, 5 -H, *J *= 8.5 Hz), 6.79 (d, 1H, 6-H, *J *= 8.5 Hz), 6.87–7.06 (m, 4H, 3′′′-H, 4′′′-H, 5′′′-H, and 6′′′-H Ar-OMe), 8.14 (s, 1H, 3-H); ^13^C-NMR (CDCl_3_): δ 36.8, 50.6, 53.4, 55.4, 55.8, 56.1, 104.6, 107.4, 111.2, 118.2, 120.0, 123.0, 126.5, 131.5, 133.4, 141.3, 143.0, 148.6, 150.8, 152.3, 193.5; HRMS (EI) Calcd for (M^+^): C_24_H_28_N_2_O_4_S 440.17698, found: 440.17541.

*1-(4,7-Dimethoxybenzo[b]thiophen-2-yl)-3-(4-pyridin-2-yl)-piperazin-1-yl)-1-propanone* (**6e**). Prepared from **5a** (70 mg, 0.28 mmol) and 1-(pyridin-2-yl) piperazine (73 mg, 0.45 mmol). Quantitative crude yield. Purified by recrystallization (EtOH) as yellow pale crystals (66 mg, 60%); mp 174–175 °C; IR_νmax_ (KBr): 3032 (C-H Ar), 2938 (C-H Aliph.), 1656 (C=O), 1594 (Ar, C=C) cm^−1^; ^1^H-NMR (CDCl_3_): δ 2.64 (t, 4H, 2′′-H and 6′′-H, *J *= 5.0 Hz), 2.92 (t, 2H, 3′-H, *J* = 7.5 Hz), 3.26 (t, 2H, 2′-H, *J* = 7.5 Hz), 3.55 (t, 4H, 3′′-H and 5′′-H; *J *= 5.0 Hz), 3.93 (s, 3H, Ar-OMe C-4 or C-7), 3.95 (s, 3H, Ar-OMe C-7 or C-4), 6.59-6.68 (m, 3H, 5-H, 4′′′-H, 6′′′-H), 6.79 (d, 1H, 6-H, *J *= 8.5 Hz), 7.5 (td, 1H, 5′′′-H, *Jo* = 7.6, *J_m_* = 2.2 Hz), 8.14 (s, 1H, 3-H), 8.18 (d, 1H, 3′′′-H, *J *= 5.2 Hz); ^13^C-NMR (75 MHz, CDCl_3_): δ 36.8, 45.2, 53.1, 53.4, 55.8, 56.1, 104.7, 107.1, 107.4, 113.3, 126.5, 131.5, 133.4, 137.4, 142.9, 147.9, 148.6, 150.8, 159.5, 193.3; HRMS (EI) Calcd for (M^+^): C_22_H_25_N_3_O_3_S 411.16166, found: 411.15976.

*1-(4,7-Dimethoxybenzo[b]thiophen-2-yl)-3-[4-(4-nitrophenyl)piperazin-1-yl]-1-propanone* (**6f**). Prepared from **5a** (72 mg, 0.29 mmol) and 1-(4-nitrophenyl) piperazine (88mg, 0.43 mmol). Crude yield quantitative. Purified by recrystallization (EtOH/CH_3_CN 5:1) as yellow crystals (82 mg, 65%); mp 161–162 °C; IR_νmax_ (KBr): 3030 (C-H Ar), 2934 (C-H Aliph.), 1647 (C=O), 1597 (NO_2_), 1326 (NO_2_) cm^−1^; ^1^H-NMR (CDCl_3_): δ 2.66 (t, 4H, 2′′-H and 6′′-H, *J *= 5.0 Hz,), 2.95 (t, 2H, 3′-H, *J* = 7.1 Hz), 3.24 (t, 2H, 2′-H, *J* = 7.1 Hz), 3.41 (t, 4H, 3′′-H and 5′′-H, *J *= 5.0 Hz), 3.93 (s, 3H, Ar-OMe C-4 or C-7), 3.95 (s, 3H, Ar-OMe C-7 or C-4), 6.67 (d,1H, 5-H, *J *= 8.5 Hz), 6.79 (d, 3H, 6-H, and 2′′′-H, 6′′′-H Ar-NO_2_, *J *= 8.8 Hz), 8.1 (d, 2H, 3′′′-H and 5′′′-H, Ar-NO_2_, *J *= 9.3 Hz), 8.13 (s,1H, 3-H); ^13^C-NMR (CDCl_3_): δ 36.7, 47.0, 52.6, 53.2, 55.8, 56.1, 104.7, 107.5, 112.6, 125.9, 126.5, 131.4, 133.4, 138.4, 142.8, 148.6, 150.7, 154.8, 193.1; HRMS (EI) Calcd for (M^+^): C_23_H_25_N_3_O_5_S 455.15149, found: 455.15128.

#### 3.2.2. Synthesis of *1-(Benzo[b]thiophen-2-yl)-3-(4-arylpiperazin-1-yl)-1-propanone Derivatives* **7a–f**

*1-Benzo[b]thiophen-2-yl-2-propen-1-one *(**5b**) [14]. To a solution of alcohol **4b** (430 mg, 2.28 mmol) in CH_2_Cl_2_ (50 mL), was added MnO_2_ (991 mg, 11.4 mmol), anhydrous MgSO_4_ (275 mg, 2.28 mmol), and the mixture stirred, at r.t for 6 h. The crude residue was then filtered and concentrated *in vacuo*, to afford the crude product (321 mg), which was purified by column chromatography (CH_2_Cl_2_) to provide pure **5b** (265 mg, 62%); mp 45–46 °C; IR_νmax_ (KBr): 3032 (C-H Arom.), 1661 (C=O), 1601 (C=C); ^1^H-NMR (CDCl_3_): δ 5.92 (dd,1H, 3′-H_cys_, *J_cys_* = 10.4 Hz; *J_gem_* = 1.5 Hz), 6.54 (dd, 1H, 3′-H trans, *J_trans_* = 17.0 Hz; *J_gem_* = 1.5 Hz), 7.18 (dd, 1H, 2′-H, *J_trans_* = 17.0 Hz, *J_cys_* = 10.4 Hz), 7.41 (td, 1H, 6-H or 5-H, *J_o_* = 7.5 Hz; *J_m_* = 1.25 Hz), 7.47 (td, 1H, 5-H or 6-H, *J_o_* = 7.5 Hz; *J_m_* = 1.25 Hz), 7.87 (t, 2H, 4-H and 7-H, *J_o_* = 7.1 Hz), 8.0 (s, 1H, 3-H); ^13^C-NMR (CDCl_3_): δ 123.0, 125.3, 126.1, 127.6, 128.7, 128.8, 131.4, 139.2, 142.8, 144.2, 183.7; HRMS (EI) Calcd for C_11_H_8_OS (M+): 188.02959, found: 188.02904.

*1-(Benzo[b]thiophen-2-yl)-3-(4-phenylpiperazin-1-yl)-1-propanone *(**7a**). To a solution of **5b** (131 mg, 0.71 mmol) and 1-phenylpiperazine (115 mg, 0.71 mmol), in dichloromethane (20 mL) was added the inorganic supported reagent SiO_2_–MnO_2_ (4:1) (2.0 g) and the suspension was vigorously stirred for 15 min at room temperature. The solvent was removed *in vacuo* and the solid was irradiated at 900 W for 15 min, until TLC showed that the starting product had disappeared. The solid was thoroughly washed with AcOEt followed by removal of the solvent to afford **7a** as a crude in quantitative yield. The solid residue was purified by column chromatography (AcOEt) to give a pale yellow oil (145 mg, 59%); IR_νmax._ (KBr): 2917 (C-H Arom.), 2849 (C-H aliphatic), 1655 (C=O), 1599 (C=C) cm^−1^; ^1^H-NMR (CDCl_3_): δ 2.77 (t, 4H, 2′′-H and 6′′-H, *J* = 4.8 Hz); 3.01 (t, 2H, 3′-H, *J* = 7.2 Hz); 3.27 (t, 4H, 3′′-H and 5′′-H, *J *= 4.8 Hz); 3.32 (t, 2H, 2′-H, *J *= 7.2 Hz), 6.88 (t, 1H, 4′′′-H, *J *= 9.2 Hz); 6.97 (d, 2H, 2′′′-H and 6′′′-H, *J *= 8.0 Hz); 7.30 (t, 2H, 3′′′-H and 5′′′-H, *J *= 9.6 Hz); 7.46 (t, 2H, 6-H or 5-H, *J* = 6.8 Hz); 7.53 (t, 2H, 5-H or 6-H, *J *= 6.8 Hz); 7.94 (t, 2H, 4-H and 7-H, *J* = 8.4 Hz); 8.05 (s, 1H, 3-H); ^13^C-NMR (CDCl_3_) δ: 32.5; 49.1 (2C); 53.7 (2C); 56.5; 113.2 (2C); 116.1; 119.8; 123.0; 125.1; 127.5; 129.1 (2C); 138.4; 193.8; HRMS (EI) Calcd for (M^+^): 350.14528, found: 350.14605.

*3-[4-(2-Fluorophenyl)piperazin-1-yl]-1-(benzo[b]thiophen-2-yl)-1-propanone *(**7b**). To a solution of **5b** (59 mg, 0.32 mmol) and 1-(2-fluorophenyl)piperazine (57 mg, 0.32 mmol), in dichloromethane (20 mL) was added the inorganic supported reagent SiO_2_–MnO_2_ (4:1, 2.0 g). After solvent removal the solid mixture was irradiated at 900 W for 10 min. Purification using a chromatographic column afforded 105 mg, (90%) of pure **7b** as a yellow oil. IR_νmax_ (KBr): 3040 (C-H arom.), 2822 (C-H aliph.), 1661 (C=O), 1501 (C=C) cm^−1^; ^1^H-NMR (CDCl_3_) δ: 2.69 (m, 4H, 2′′-H and 6′′-H); 2.92 (t, 2H, 3′-H, *J *= 6.8 Hz), 3.06 (m, 4H, 3′′-H and 5′′-H), 3.20 (t, 2H, 2′-H, *J *= 6.8 Hz), 6.85–7.02 (m, 4H, 3′′′-H and 6′′′-H), 7.35 (t, 1H, 5-H or 6-H, *J *= 7.2 Hz), 7.41 (t, 1H, 6-H or 5-H, *J *= 7.2 Hz); 7.81 (d, 1H, 7-H or 4-H, *J *= 8.0Hz); 7.85 (d, 1H, 4-H or 7-H, *J* = 8.0Hz); 7.98 (s, 1H, 3-H); ^13^C-NMR (CDCl_3_) δ: 36.8; 52.2 (2C); 53.1 (2C); 53.6; 113.7 (d, ^2^*J *= 49 Hz); 118.4 (d, ^3′^*J* = 2.8 Hz); 122.9 (d, ^2′^*J *= 7.0 Hz); 124.4, 125.5 (d, ^4^*J *= 3,5 Hz);128.6 (d, ^3^*J *= 66 Hz); 130.8; 132.9, 136.2, 138.0, 138.1, 142.2, 147.5, 158.0 (d, ^1^*J *= 240 Hz), 192,3; HRMS (EI) Calcd for C_21_H_21_FN_2_OS (M^+^): 368.13586, found: 368.13640.

*3-[4-(4-Fluorophenyl)piperazin-1-yl]-1-(benzo[b]thiophen-2-yl)-1-propanone *(**7c**). To a solution of **5b** (90 mg, 0.0.49 mmol) and 1-(4-fluorophenyl)piperazine (132 mg, 0.73 mmol) in dichloromethane (20 mL) a mixture of the inorganic supported reagent SiO_2_–MnO_2_ (4:1, 2.0 g) was added. After solvent removal the solid mixture was irradiated at 900 W for 10 min. Purification by recrystallization (EtOH/hexane), afforded 68 mg. (38%) of pure **7c**; mp 117–118 °C; IR_νmax _(KBr): 2954 (C-H arom.), 2822 (C-H aliph.), 1660 (C=O), 1510 (C=C) cm^−1^; ^1^H-NMR (CDCl_3_) δ: 2.70 (t, 4H, 2′′-H and 6′′-H, *J *= 4.8 Hz); 2.95 (t, 2H, 3′-H, *J *= 7.4 Hz); 3.13 (t, 4H, 3′′-H and 5′′-H, *J *= 4.7 Hz); 3.26 (t, 2H, 2′-H, *J *= 7.4 Hz); 6.87 (dd , 2H, 2′′′-H and 6′′′-H, *J *= 4.6 Hz); 6.96 (t, 2H, 3′′′-H and 5′′′-H, *J *= 9.0 Hz); 7.45 (m, 2H, 5-H and 6-H, *J *= 1.4 Hz, *J *= 9.4 Hz); 7.89 (t, 2H, 4-H and 7-H, *J *= 7.0 Hz); 8.00 (s, 1H, 3-H); ^13^C-NMR (CDCl_3_) δ: 40.1; 53.3 (2C); 56.3 (2C); 118.5; 118.8; 121.0 (d, 2C, ^3^*J* = 7.6 Hz); 126.2; 128.2; 129.1; 130.7; 132.2; 142.2; 145.7; 146.7; 151.0; 158.7; 161.9 (d, ^1^*J* = 239 Hz); 196.5; HRMS (EI) Calcd for C_21_H_21_FN_2_OS (M^+^): 368.13586, found: 368.13750.

*3-[4-(2-Methoxyphenyl)piperazin-1-yl]-1-(benzo[b]thiophen-2-yl)-1-propanone* (**7d**). To a solution of **5b** (77 mg, 0.42 mmol) and 1-(2-methoxyphenyl)piperazine (80 mg,0.42 mmol), in dichloromethane (20 mL) the inorganic supported reagent SiO_2_-MnO_2_ (4:1, 2.0 g) was added. After solvent removal, the solid mixture was irradiated at 900 W for 10 min. Purification by recrystallization (EtOH/hexane), afforded 78 mg. (50%) of pure **7d**; mp: 45–47 °C; IR_νmax_. (KBr): 2918 (C-H Arom), 2817 (C-H aliph.), 1662 (C=O), 1594 (C=C); ^1^H-NMR (CDCl_3_): 2.70 (m 4H, 2′′-H and 6′′-H); 2.91 (t, 2H, 3′-H, *J *= 7.6 Hz); 3.10 (m, 4H, 3′′-H and 5′′-H), 3.21 (t, 2H, 2′-H, *J *= 7.6 Hz); 3.79 (s, 3H, 2′′′-OCH_3_); 6.78–6.95 (m, 4H, 3′′′-H and 6′′′-H); 7.34 (t, 2H, 6-H or 5-H, *J *= 8.0 Hz); 7.40 (t, 2H, 5-H or 6-H, *J *=.8.0 Hz); 7.82 (t, 2H, 4-H and 7-H, *J *= 9.2 Hz); 7.94 (s, 1H, 3-H); ^13^C-NMR (CDCl_3_) δ: 30.9; 50.8 (2C); 53.3; 53.5 (2C); 54.7; 111.3; 118.3; 121,1; 123,0; 125,0; 128,8; 129,1; 137,7; 138,1; 139,1; 139,2; 152.3; 193.4; HRMS (EI) Calcd for C_22_H_24_N_2_O_2_S (M^+^): 380.15585, found: 380.15460.

*1-(Benzo[b]thiophen-2-yl)-3-(4-pyridin-2-yl)-piperazin-1-yl)-1-propanone* (**7e**). To a solution of **5b** (122 mg, 0.66 mmol) and 1-(2-methoxyphenyl)piperazine (162 mg, 0.99 mmol), in dichloromethane (20 mL) the inorganic supported reagent SiO_2_-MnO_2_ (4:1; 2.0 g) was added. After solvent removal, the solid mixture was irradiated at 900 W for 10 min. Purification by column chromatography (AcOEt) afforded pure **7e** (163 mg, 71%); mp: 56–58 °C; IR_νmax _(KBr): 3030 (C-H Ar), 2964, (C-H Aliph.), 1661 (C=O), 1592 (Ar, C=C)cm^−1^; ^1^H-NMR (CDCl_3_) δ: 2.65 (t, 4H, 2′′-H and 6′′-H, *J *= 4.9 Hz); 2.93 (t, 2H, 3′-H, *J *= 7.4 Hz); 3.27 (t, 2H, 2′-H, *J *= 7.3 Hz); 3.56 (t, 4H, 3′′-H and 5′′-H, *J *= 4.9 Hz); 6.62 (m, 2H, 4′′′-H and 6′′′-H, *J *= 5.0 Hz); 7.45 (m, 3H, 5-H, 6-H and 5′′′-H); 7.88 (t, 2H, 4-H and 7-H, *J *= 7.8 Hz); 8.00 (s, 1H, 3-H); 8.19 (m, 1H, 3′′′-H); ^13^C-NMR (CDCl_3_) δ: 36.9; 45.2 (2C); 53.0 (2C); 53.3; 107.1; 113.4; 123.0; 125.0; 126.0; 127.5; 129.1; 137.5; 139.1; 142.5; 143.6; 147.9; 159.4; 193.4. HRMS (EI) Calcd for (M^+^): C_20_H_21_N_3_OS: 351.14053; found: 351.14090.

*1-(Benzo[b]thiophen-2-yl)-3-[4-(4-nitrophenyl)piperazin-1-yl]-1-propanone* (**7f**).To a solution of **5b** (90 mg, 0.49 mmol) and 1-(4-nitrophenyl)piperazine (152 mg, 0.73 mmol), in dichloromethane (20 mL) was added the inorganic supported reagent. The crude compound was purified by recrystallization (ethanol/hexane 4:1), to afford 131 mg (69%) of pure **7f**; mp: 138–141 °C; IR_νmax _(KBr): 2949 (C-H arom.), 2833 (C-H aliph.), 1656 (C=O), 1601 (C=C), 1330 (NO_2_) cm^−1^; ^1^H-NMR (CDCl_3_) δ: 2.68 (t, 4H, 2′′-H and 6′′-H, *J = *5.2 Hz), 2.96 (t, 2H, 3-H, *J = * 7.1 Hz), 3.26 (t, 2H, 2′-H, *J *= 7.1 Hz), 3.43 (t, 4H, 3′′-H and 5′′-H, *J *= 5.2 Hz), 6.82 (dd, 2H, 2′′′-H and 6′′′-H, *J*_m _= 2.1 Hz, *J*_o _= 8.4 Hz), 7.46 (m, 2H, 5-H and 6-H, *J *= 1.2 Hz, *J *= 3.4 Hz), 7.90 (t, 2H, 4-H and 7-H, *J *= 7.0 Hz), 8.00 (s, 1H, 3-H), 8.12 (dd, 2H, 3′′′-H and 5′′′-H, *J *= 2.0 Hz, *J *= 7.4 Hz); ^13^C-NMR (CDCl_3_) δ: 40.0, 50.1 (2C), 55.8 (2C), 56.2, 115.8 (2C), 126.2 (2C), 128.3, 129.1, 129.1, 130.7, 132.3, 141.6, 142.2, 145.6, 146.6, 157.9, 196.3; HRMS (EI) Calcd for C_21_H_21_NO_3_S (M^+^): 395.13037, found: 395.13237.

#### 3.2.3. General Procedure for the Preparation of *1-(4,7-Dimethoxybenzo[b]thiophen-2-yl)-3-(4-arylpiperazin-1-yl)-1-propanol Derivatives* **8a–f**

*1-(4,7-Dimethoxybenzo[b]thiophen-2-yl)-3-(4-phenyl-piperazin-1-yl)-1-propanol* (**8a**). To a solution of *1-(4,7-dimethoxybenzo[b]thiophen-2-yl)-3-(4-phenylpiperazin-1-yl)-1-propanone* (**7a**, 144 mg, 0.35 mmol) in methanol (20 mL), NaBH_4_ (57 mg, 1.5 mmol) was added and the mixture was vigorously stirred for 90 min at room temperature, after which the mixture was diluted with water (50 mL) and extracted with AcOEt (30 mL × 3). The combined organic layers were dried (Na_2_SO_4_), and removal of the solvent afforded 142 mg of a crude, which was further purified by recrystallization (EtOH/petroleum ether 2:1) to provide pure **8a** (99 mg, 68.3% yield) as yellow pale crystals; mp 148–149 °C; IR_νmax. _(KBr): 3425(O-H), 3032 (C-H Ar), 2825 (C-H Aliph.), 1601 (C=C Ar) cm^−1^; ^1^H-NMR (CDCl_3_): δ 1.46–1.75 (bs,1H, OH), 1.96–2.2 (m, 2H, 2′-H), 2.65–2.83 (m, 6H, 3′-H and 2′′-H and 6′′-H), 3.23 (t, 4H, 3′′-H, and 5′′-H, *J* = 4.8 Hz), 3.90 (s, 3H, Ar-OMe,C-7), 3.94 (s, 3H, Ar-OMe, C-4), 5.29 (m, 1H, 1′-H), 6.63 (d, 1H, 5-H, *J* = 8.3 Hz), 6.65 (d, 1H, 6-H, *J* = 8.3 Hz), 6.82–7.11 (m, 4H, 2′′′-H, 4′′-H, 6′′-H, and OH), 7.26–7.33 (m, 3H, 3-H, 3′′′- and 5′′′-H); ^13^C-NMR (CDCl_3_): δ 33.3 (C-2), 49.2 (C-3′ and C-5′-Pip), 53.2 (C-2′ and C-6′-Pip), 55.9 (ArOMe, C-4′ or C-7′), 56.0 (ArOMe, C-7′ or C-4′), 56.4 (C-3), 72.3 (C-1), 103.9(C-5′_BT_), 104.6(C-6′_BT_), 115.9 (C-3′_BT_), 116.3 (C-2′′ and C-6′′-Ph), 120.1 (C-4′′-Ph), 129.2 (C-3′′ and C-5′′-Ph), 129.2 (C-7a), 132.0 (C-3a), 149.1 (C-4′_BT_), 149.5 (C-2′_BT_ or C-7′_BT_), 149.7 (C-7′_BT_ or C-2′_BT_), 151.1 (C-1′′-Ph); HRMS (EI) Calcd for C_23_H_28_N_2_O_3_S 412.18206 (M^+^), found: 412.18115.

*3-[4-(2-Fluorophenyl)piperazin-1-yl]-1-(4,7-dimethoxybenzo[b]thiophen-2-yl)-1-propanol* (**8b**). Prepared from **7b** (175 mg, 0.41 mmol) and sodium borohydride (46 mg, 1.20 mmol). The crude was purified by preparative chromatography (EtOH) to afford 111 mg (63%) of the title compound; mp 128–129 °C; IR_νmax _(KBr): 3422 (O-H), 2953 (C-H Aliph.), 1511 (ArC=C), 1485 (Ar C=C) cm^−1^; ^1^H-NMR (CDCl_3_): δ 1.35–1.82 (bs, 1H, OH), 1.97–2.22 (m, 2H, 2′-H), 2.76 (m, 6H, 2′′-H, 6′′-H and 3′-H), 3.14 (m, 4H, 3′′-H and 5′′-H), 3.90 (s, 3H, Ar-OMe), 3.94 (s, 3H, Ar-OMe), 5.27 (m, 1H, 1′-H), 6.63 (d, 1H, 5-H, *J *= 8.6 Hz), 6.67 (d, 1H, 6-H, *J *= 8.6 Hz), 6.91–7.12 (m, 4H, 3′′′-H, 4′′′-H, 5′′′-H, and 6′′′-H, Ar-F), 7.31 (s, 1H, 3-H); ^13^C-NMR (CDCl_3_): δ 33.2 (C-2), 50.6 (C-3′ and C-5′-Pip), 53.3 (C-2′ and C-6′-Pip), 55.9 (ArOMe, C-4′ or C-7′), 56.0 (ArOMe, C-7′ or C-4′), 56.4 (C-3), 72.2 (C-1), 103.9 (C-5′_BT_), 104.6 (C-6′_BT_), 115.9 (C-3′_BT_), 116.1 (d, C-3′′, *^2^J_C-F_* = 21.0 Hz ), 119.0 (d, C-6′′, *^3′^J* = 3.0 Hz), 122.7 (d, C-1′′, *^2′^J_C-F_* = 8.0 Hz), 124.6 (d, C-5′′, *^4^J_C-F_* = 3.8 Hz), 129.1 (C-7a), 132.0 (C-3a), 139.8 (d, C-4′′, *^3^J* = 8.6 Hz), 148.8 (C-4′_BT_), 149.1 (C-2′_BT_ or C-7′_BT_), 149.6 (C-7′_BT_ or C-2′_BT_), 155.7 (d, C-2′′, *^1^J_C-F_* = 245.0 Hz); HRMS (EI) Calcd for C_23_H_27_FN_2_O_3_S 430.17264 (M^+^), found: 430.17010.

*3-[4-(4-Fluorophenyl)piperazin-1-yl]-1-(4,7-dimethoxy-benzo[b]thiophen-2-yl)-1-propanol* (**8c**). Prepared from 3-[4-(4fluorophenyl)piperazin-1-yl]-1-(4,7-dimethoxybenzo[b]thiophen-2-yl)-1-propanone (**7c**, 50.0 mg, 0.116 mmol) and sodium borohydride (15 mg, 0.39 mmol). Crude yield quantitative. The compound was purified by preparative chromatography on silica gel (EtOH) (40 mg, 79.7%); mp 55–56 °C; IR ν_max_ (KBr): 3419 (O-H), 2919 (C-H Aliph.), 1510 (ArC=C), 1485 (Ar C=C) cm^−1^; ^1^H-NMR (CDCl_3_): δ 1.50–1.72 (bs,1H, OH), 1.97–2.22 (m, 2H, 2′-H), 2.75 (m, 6H, 2′′-H and 6′′-H and 3′-H), 3.16 (t, 4H, 3′′-H and 5′′-H; *J *= 4.8 Hz), 3.91 (s, 3H, Ar-OMe), 3.95 (s, 3H, Ar-OMe), 5.27 (m, 1H, 1′-H), 6.63 (d, 1H, 5-H, *J *= 8.6 Hz), 6.67 (d, 1H, 6-H, *J *= 8.6 Hz), 6.84–6.90 (m, 2H, 2′′′-H and 6′′′-H Ar-F), 6.94–7.05 (m, 2H, 3′′′-H and 5′′′-H, Ar-F), 7.30 (d, 1H, 3-H, *J* = 1.1 Hz); ^13^C-NMR (CDCl_3_): δ 33.6 (C-2), 50.7 (C-3′-Pip and C-5′-Pip), 53.6 (C-2′ and C-6′-Pip), 56.2 (C-3), 56.4 (ArOMe, C-4′ or C-7′), 56.7 (ArOMe, C-7′ or C-4′), 72.6 (C-1), 104.2 (C-5′_BT_), 104.9 (C-6′_BT_), 115.9 (C-3′-_BT_), 116.2 (d, C-2′′ and C-6′′, *^2^J_C-F_* = 11.3 Hz), 118.5 (d, C-3′′ and C-5′′, *^3^J_C-F_* = 7.6 Hz), 129.8 (C-7a), 132.4 (C-4a), 148.0 (d, C-1′′, *^4^J_C-F_* = 1.5 Hz), 149.1 (C-4′_BT_), 149.4 (C-2′_BT_), 150.0 (C-7′_BT_), 157.8 (d, C-4′′, *^1^J_C-F_* = 239 Hz); HRMS (EI) Calcd for C_23_H_27_FN_2_O_3_S 430.17264 (M^+^), found: 430.17051.

*1-(4,7-Dimethoxy-benzo[b]thiophen-2-yl)-3-[4-(2-methoxyphenyl)piperazin-1-yl]-1-propanol* (**8d**). Prepared from 1-(4,7-dimethoxybenzo[b]thiophen-2-yl)-3-[4-(2-methoxyphenyl)piperazin-1-yl]-1-propanone (**7d**, 80 mg, 0.18 mmol) and sodium borohydride (207 mg, 5.4 mmol), in methanol (20 mL). Crude yield quantitative. The crude was purified by preparative chromatography (EtOH) to afford 75 mg of the tile compound (93.4%) as pale yellow crystals; mp 55–56 °C; IR_νmax. _(KBr): 3415 (O-H), 3029 (C-H Ar), 2934 (C-H Aliph.), 1257 (C-O) cm^−1^; ^1^H-NMR (CDCl_3_): δ 1.52–1.70 (bs, 1H, OH), 1.95–2.2 (m, 2H, 2′-H), 2.65–2.92 (m, 6H, 3-H and 2′′-H and 6′′-H), 3.12 (m, 4H, 3′′-H, 5′′-H), 3.86 (s, 3H, 2′′′-Ar-OMe), 3.90 (s, 3H, Ar-OMe, C-7), 3.94 (s, 3H, Ar-OMe, C-4), 5.28 (m, 1H, 1′-H), 6.63 (d, 1H, 5-H, *J* = 8.3 Hz), 6.67 (d, 1H, 6-H, *J *= 8.3 Hz), 6.85–7.06 (m, 4H, 3′′′-H, 4′′′-H, 5′′′-H, and 6′′′-H Ar-OMe), 7.31 (d, 1H, 3-H *J* = 1.1 Hz); ^13^C-NMR (CDCl_3_): δ 33.1 (C-2), 50.6 (C-3′ and C-5′-Pip), 53.3 (C-2′ and C-6′-Pip), 55.3 (ArOMe), 55.8 (ArOMe, C-4′ or C-7′), 55.9 (ArOMe, C-7′ or C-4′), 56.5 (C-3), 72.7 (C-1), 103.7 (C-5′_BT_), 104.4 (C-6′_BT_), 111.0 (C-3′′), 115.8 (C-3′_BT_), 118.3 (C-6′′), 121.0 (C-5′′), 123.2 (C-4′′), 129.4 (C-7a), 132.0 (C-3a), 140.9 (C-1′′), 148.7 (C-2′_BT_), 149.0 (C-4′_BT_), 149.8 (C-2′′), 152.2 (C-7′_BT_); HRMS (EI) Calcd for C_24_H_30_N_2_O_4_S 442.19263 (M^+^), found: 442.19009.

*1-(4,7-Dimethoxybenzo[b]thiophen-2-yl)-3-(4-pyridin -2-yl)-piperazin-1-yl)-1-propanol* (**8e**). Prepared from 1-(4,7-dimethoxybenzo[b]thiophen-2-yl)-3-(4-pyridin-2-yl)-piperazin-1-yl)-1-propanone (**7e**, 117 mg, 0.28 mmol) and sodium borohydride (40 mg, 1.05 mmol). Quantitative yield. The crude was purified by preparative chromatography (EtOH) to afford 90 mg (76.6%) of pure **8e**; mp 157–158 °C; IR_νmax_. (KBr): 3422 (O-H), 2832 (C-H Aliph.), 1596 (ArC=N), 1485 (Ar C=C) cm^−1^; ^1^H-NMR (CDCl_3_): 1.49–1.71 (bs, 1H, OH), 1.92–2.25 (m, 2H, 2′-H), 2.6–2.8 (m, 6H, 2′′-H and 6′′-H, 3′-H), 3.47 (m, 4H, 3′′-H and 5′′-H, ), 3.90 (s, 3H, Ar-OMe), 3.95 (s, 3H, Ar-OMe), 5.28 (m, 1H, 1′-H), 6.63–6.67 (m, 4H, 5-H, 6-H and 3′′′-H, 5′′′-H), 7.31 (s, 1H, 3-H), 7.49 (td, 1H, 4′′′-H, *Jo* = 6.7 Hz, *Jm *= 2.0 Hz), 8.2 (dd, 1H, 6′′′-H, *Jo* = 5.4 Hz, *Jm *= 2.0 Hz); ^13^C-NMR (CDCl_3_): δ 33.2(C-2), 45.2 (C-3′ and C-5′-Pip), 53.0 (C-2′ and C-6′-Pip), 55.8 (ArOMe, C-4′ or C-7′), 56.0 (ArOMe, C-7′ or C-4′), 56.5 (C-3), 72.2 (C-1), 103.8 (C-5′_BT_), 104.5 (C-6′_BT_), 107.1 (C-3′′),113.3 (C-5′′),115.9 (C-3′_BT_), 129.3 (C-7a), 132.0 (C-3a), 137.6 (C-4′′), 148.0 (C-6′′), 148.8 (C-2′_BT_), 149.0 (C-4′_BT_), 149.7 (C-7′_BT_), 159.3 (C-2′′); HRMS (EI) Calcd for C_22_H_27_N_3_O_3_S 413.17731 (M^+^), found: 413.17593.

*1-(4,7-Dimethoxybenzo[b]thiophen-2-yl)-3-[4-(4-nitrophenyl)piperazin-1-yl]-1-propanol* (**8f**). Prepared from 1-(4,7-dimethoxybenzo[b]thiophen-2-yl)-3-[4-(4-nitrophenyl)piperazin-1-yl]-1-propanone (**7f**, 58 mg, 0.127 mmol) and sodium borohydride (19 mg, 0.5 mmol). Quantitative yield. Purified by preparative chromatography (EtOH) to afford 42 mg (72%) of pure **8f**; mp 173–174 °C; IR_νmax _(KBr): 3420 (O-H), 3030 (C-H Ar), 2918 (C-H Aliph.), 1596 (NO_2_), 1327 (NO_2_), 1487 (C=C Ar) cm^−1^;^1^H-NMR (CDCl_3_): δ 1.52–1.72 (bs, 1H, OH), 1.95–2.22 (m, 2H, 2′-H), 2.72 (m, 6H, 2′′-H, 6′′′-H and 3-H), 3.47 (t, 4H, 3′′-H and 5′′-H; *J *= 5.0 Hz), 3.93 (s, 3H, Ar-OMe), 3.97 (s, 3H, Ar-OMe), 5.26 (m, 1H, 1′-H), 6.63 (d, 1H, 5-H, *J *= 8.8 Hz), 6.66 (d, 1H, 6-H, *J *= 8.8 Hz), 6.84 (d, 2H, 2′′′-H Ar-NO_2_ and 6′′′-H Ar-NO_2_, *J *= 9.4 Hz), 7.33 (s, 1H, 3-H), 8.23 (d, 2H, 3′′′-H Ar-NO_2_ and 5′′′-H, Ar-NO_2_, *J *= 9.4 Hz); ^13^C-NMR (CDCl_3_): δ 33.7 (C-2), 47.4 (C-3′, C-5′-Pip), 53.0 (C-2′,C-6′-Pip), 56.2 (ArOMe, C-4′ or C-7′), 56.4 (ArOMe, C-7′ or C-4′), 56.6 (C-3), 72.4 (C-1), 104.4 (C-5′_BT_), 105.0 (C-6′_BT_), 113.3 (C-2′′ and C-6′′ Ar-NO_2_), 116.5 (C-3′_BT_), 126.3 (C-3′′ and C-5′′ Ar-NO_2_), 129.8 (C-7a), 132.4 (C-3a), 139.2 (C-4′′-Ar-NO_2_), 149.1 (C-4′_BT_), 149.5 (C-2′_BT_ or C-7′_BT_), 149.6 (C-7′_BT_ or C-2′_BT_), 155.1 (C-1′′-Ar-NO_2_); HRMS (EI) Calcd for C_23_H_27_N_3_O_5_S 457.16714 (M^+^), found: 457.16591.

### 3.3. Biological Methods

#### Radioligand Binding Assays

For binding assays, male Sprague-Dawley rats (*Rattus norvegicus albinus*) weighing about 200–250 g were killed by decapitation, and their brains were rapidly removed and dissected (cerebral cortex for 5-HT_1A_R). Binding assays were performed by a modification of the procedure previously described by Srinivas *et al*. [27] The cerebral cortex was homogenized in about 10 volumes of ice-cold 50 mM Tris-HCl buffer (pH 7.4) containing 0.32 M sucrose using a glass-teflon homogenizer and an Ultra Turrax T8 IKA Labortechnik (5 × 30 s). Then the homogenate was centrifuged at 2.264 g for 10 min at 4 °C (Heraeus Sepatech Suprafuge 22 ultracentrifuge). The pellet was discarded and the supernatant was centrifuged at 56.599 g for 30 min at 4 °C. The pellet was resuspended in 20 volumes of ice-cold 50 mM Tris-HCl buffer (pH 7.4) and centrifugated again at 56.599 g for 30 min at the same temperature as before. The pellet was then washed once by resuspension in fresh buffer and centrifuged as before. Finally the pellet was splitted and frozen until the day of experiment. The final pellet was resuspended in ice-cold 50 mM Tris-HCl buffer (pH 7.4). Fractions of 50 μL of the final membrane suspension (about 127 μg of protein) were incubated at 37 °C for 30 min with 5 nM [^3^H]-8-OH-DPAT (170 Ci/mmol), in the presence or absence of the competing drug, in a final volume of 200 μL of assay. Nonspecific binding was determined with 10 μM 8-OH-DPAT. All incubations were terminated by the addition of 2 mL of ice-cold Tris-HCl buffer (pH 7.4). The reaction mixture was rapidly filtrated through GF/C filters (pre-soaked in buffer containing 0.3% polyethyleneimine) under vacuum and washed twice with 2 mL of ice-cold buffer. Filters were dried and transferred to scintillation vials containing 4 mL of scintillation cocktail (PPO 4 g/L; POPOP 0.1 g/L in toluene) and allowed to equilibrate overnight before counting in a liquid scintillation counter at 28% efficiency.

### 3.4. Molecular Modeling

#### 3.4.1. 5.HT_1A_ Receptor Modeling

The aminoacid sequence for the human 5-HT_1A_R was obtained from SWISS-PROT [28] (access number: P08908). Moreover, as a template for comparative modeling was used three-dimensional crystal structure of bovine rhodopsin obtained from the Protein Data Bank (www.rcsb.org/pdb) (PDB code: 2R4R) [29]. The initial alignment between *2R4R* and the sequence of 5-HT_1A_ were made with the program Clustal W [30].

The models were generated using the program MODELLER [31]. MODELLER produces comparative models that meet space restrictions with simultaneous optimization of energy CHARMM [32]. The shape of space constraints was obtained by statistical analysis of the relationship between pairs of homologous structures from a database of sequence alignments of 416 known 3D structures of proteins in 105 families. The comparative models were verified by validation programs PROCHECK [33,34]. The ligands were optimized to get its lowest energy conformation with the program SPARTAN 2. In all cases the ligands were protonated on the aliphatic nitrogen atom in the piperazine ring.

#### 3.4.2. Molecular Docking

The grids of the maps that represent the 5-HT_1A_R in the docking process were calculated with AutoGrid (included in the program AutoDock) [35]. The grids were chosen large enough to include not only the active site but significant portions of the surrounding area. The points of the grids were therefore 60 × 60 × 60 Å for the 5-HT_1A_R with a grid spacing of 0.375 Å (about one quarter of the length of a carbon-carbon bond) and as is known the importance of D116, the cubic grids were centered on the ligand binding site. The automated docking studies were carried out using the program AutoDock 3.0.5. Lamarckian genetic algorithms were applied to model the interaction between the 5-HT_1A_R and the residue D116. For the local search were used pseudo-Solis and Wets algorithms. For all docking parameters, were used standard values as described above, except the number of independent docking runs for each docking simulation. To find aspects of computing time and data size on the one hand, and convergence criteria and statistical significance on the other, 200 independent docking runs were performed for each case. The cluster analysis was performed on the docking results using a tolerance for half-square deviation of 0.5 Å. All calculations were performed in a PC Cluster Intel Pentium 4 3.0 GHz operating system that runs on Red Hat Enterprise Linux version 4.

#### 3.4.3. Binding Model Analysis

To select the most likely conformations of the complexes given by AutoDock were used quantitative and qualitative considerations. First, was elected the conformation with the lowest docking energy as a starting point. Then the chosen conformation was analyzed qualitatively based on the orientation/location of the serotonin agonist in relation to the receptor. In the event that the orientation/location of the chosen conformation did not agree with the corresponding model of agonist binding of 5-HT_1A_R, the docked complex was discarded and analyzed the next conformation with the lowest final docking energy. This procedure was repeated until they find a shape in harmony with the appropriate mode of binding of 5-HT.

## 4. Conclusions

A successful strategy for the synthesis of novel functionalized 3-[(4-aryl)piperazin-1-yl]arylpropan-1-one **6a–f** and **7a–f** along with the reduced derivative series **8a–f** is reported. The series **6a–f** and **7a–f** were obtained using microwave Michael addition of commercial arylpiperazines on the corresponding benzo[*b*]thiophenenones **5a**,**b**. The series **6a–f** displayed lower affinity values for the 5-HT_1A_ receptor, in comparison with their corresponding reduced derivatives **8a–f**. On the other hand, the **7a–f** family exhibited better activities compared to compounds **6** and **8a–f**.

As it was expected, the 2-methoxy derivatives **6d**, **8d**, **7d** ranked among the four best results obtained, along with compound **7e**. On the contrary, the *para*-substitution exemplified by the fluoro**-** and nitro**-6c**,**f**, **7f**, and **8f** apparently cause repulsive interactions in the binding site according to our docking results, confirming the detrimental effect expected for *para*-substituents on the phenyl ring of the arylpiperazine moiety. Regards to the 4,7-dimethoxy substitution pattern on the benzo[*b*]thiophene ring, we concluded that this disubstitution might be relevant in order to explain the poor activity displayed by these compounds. The more promising compound **7e**, having a *Ki* value of 2.30 μM, will help us in the design of compounds with additional modifications of future series in the exploration of potentially 5-HT_1A_ activity.
